# Supplementary material for: Hybrid Models and Biological Model Reduction with PyDSTool
Source: PLoS Comput Biol. 2012 Aug 9;8(8):e1002628. doi: 10.1371/journal.pcbi.1002628 (PMC3415397; doi:10.1371/journal.pcbi.1002628)
Supplement: Text S4 — Complete source code for the PyDSTool package (version 0.88.120504). Includes API documentation and help files linking to web pages. This file is identical to the current public release on Sourceforge.net. (ZIP) [file pcbi.1002628.s004.zip › PyDSTool/html/PyDSTool.Toolbox.optimizers.step.conjugate_gradient_step-module.html]

xml version="1.0" encoding="ascii"?


PyDSTool.Toolbox.optimizers.step.conjugate\_gradient\_step


| Home | Trees | Indices | Help | PyDSTool |
| --- | --- | --- | --- | --- |

Package PyDSTool ::
Package Toolbox ::
Package optimizers ::
Package step ::
Module conjugate\_gradient\_step

[hide private]

[frames] | no frames]

# Module conjugate\_gradient\_step

source code

Computes the conjugate gradient steps for a specific function at a
specific point


|  |  |
| --- | --- |
| Classes | [hide private] |

ConjugateGradientStep  
The basic conjugate gradient step


|  |  |
| --- | --- |
| Functions | [hide private] |

|  |  |
| --- | --- |
| CWConjugateGradientStep()  The Crowder-Wolfe or Hestenes-Stiefel conjugate gradient step | source code |

|  |  |
| --- | --- |
| DConjugateGradientStep()  The Dixon conjugate gradient step | source code |

|  |  |
| --- | --- |
| DYConjugateGradientStep()  The Dai Yan conjugate gradient step Has good convergence capabilities (same as the FR-PRP gradient) | source code |

|  |  |
| --- | --- |
| FRConjugateGradientStep()  The Fletcher Reeves conjugate gradient step Needs an exact line search for convergence or the strong Wolfe-Powell rules for an inexact line search | source code |

|  |  |
| --- | --- |
| PRPConjugateGradientStep()  The Polak-Ribiere-Polyak conjugate gradient step Can restart automatically, but needs an exact line search with a uniformely convex function to globally converge | source code |

|  |  |
| --- | --- |
| FRPRPConjugateGradientStep()  The Fletcher-Reeves modified Polak-Ribiere-Polyak conjugate gradient step Can restart automatically and has the advantages of the PRP gradient and of the FR gradient | source code |

|  |  |
| --- | --- |
| HZConjugateGradientStep()  The Hager-Zhang conjugate gradient step Has good convergence capabilities (same as the FR-PRP gradient) | source code |

| Home | Trees | Indices | Help | PyDSTool |
| --- | --- | --- | --- | --- |

|  |  |
| --- | --- |
| Generated by Epydoc 3.0.1 on Fri May 4 15:24:05 2012 | http://epydoc.sourceforge.net |
